# Heart Failure Outcomes in Primary Care: The Role of Primary Care Practice Assistants (VERAH) in German General Practice

**DOI:** 10.1155/jonm/6199599

**Published:** 2026-11-01

**Authors:** Kateryna Karimova, Anastasiya Glushan, Justyna Weronika Wünstel, Renate Klaaßen-Mielke, Catriona Friedmacher

**Affiliations:** ^1^ Institute of General Practice Goethe University Frankfurt Frankfurt 60590 Germany goethe-university-frankfurt.de; ^2^ Institute of Medical Informatics, Biometry and Epidemiology Ruhr University Bochum Bochum 44789 Germany ruhr-uni-bochum.de

**Keywords:** hospitalisation, mortality, primary care practice assistants, VERAH

## Abstract

**Background:**

Germany’s GP‐centred care (GPCC) model aims to strengthen coordinated primary care and incorporates delegated case management delivered by Primary Care Practice Assistants (VERAH). While previous studies have demonstrated beneficial effects for patients with heart failure, the additional long‐term impact of multidisciplinary primary care incorporating VERAH support compared with GPCC alone remains unclear.

**Methods:**

We conducted a retrospective cohort study using German statutory health insurance claims data from 2011 to 2022. Patients with heart failure were identified using validated ICD‐10 codes from inpatient and outpatient care records and assigned to three groups: usual care, GPCC and GP‐centred care with extended multidisciplinary team involvement (GPCC‐Plus). GPCC‐Plus was defined as a study‐specific category based on predefined VERAH‐related service codes and lump‐sum payments documented in AOK Baden‐Württemberg claims data. Outcomes included all‐cause hospitalisation, ambulatory care‐sensitive condition (ACSC) hospitalisations and all‐cause mortality. Multivariable regression models were adjusted for sociodemographic factors, comorbidities and healthcare utilisation, with time‐varying covariates included where applicable. Direct comparisons between GPCC and GPCC‐Plus were performed, and longitudinal trends were assessed over the 12‐year observation period.

**Results:**

Both GPCC models were associated with reduced hospitalisations compared with usual care, with consistently stronger effects observed in GPCC‐Plus practices involving multidisciplinary team care including Primary Care Practice Assistants (VERAH). GPCC‐Plus demonstrated the lowest odds of all‐cause hospitalisation and mortality across the study period. ACSC hospitalisations showed modest declines in both GPCC groups. Longitudinal analyses indicated sustained benefits over time, with more pronounced and consistent improvements in the GPCC‐Plus group.

**Conclusions:**

A coordinated, integrated, multidisciplinary primary care programme including delegated case management by Primary Care Practice Assistants (VERAH) is associated with sustained long‐term reductions in hospitalisations and mortality among patients with heart failure over 12 years. These findings suggest that integrating structured nonphysician workforce roles may enhance the effectiveness of GPCC beyond GP coordination alone.

**Implications for Nursing Management:**

Structured delegated case management and multidisciplinary teamwork can support continuity of care for patients with heart failure. Clear roles and communication pathways facilitate the integration of nursing and practice‐based care into GPCC.

## 1. Introduction

In high‐income countries, such as Germany and the United States, the prevalence of heart failure (HF) is expected to rise in the coming years due to population ageing and improved medical treatment [1–3]. Many HF patients are multimorbid, which increases the complexity of care and underscores the need for continuous, coordinated and comprehensive management in primary care [2]. This increasing complexity highlights the importance of structured primary care models in which general practice teams coordinate care across providers and healthcare sectors.

In Germany, routine ambulatory care does not generally require patients to pass through a primary care gatekeeper before accessing specialist care [4, 5]. Patients have unrestricted access to ambulatory specialists, often resulting in fragmented care pathways, uncoordinated specialist contacts, duplicated diagnostics and insufficient continuity of care (‘doctor shopping’), particularly among multimorbid patients with chronic conditions such as HF [6]. To strengthen the coordinating role of general practitioners (GPs), the statutory health insurance system introduced Hausarztzentrierte Versorgung (HzV; GP‐centred care [GPCC]) under §73b SGB V [7]. In HzV, participating patients select a GP as their primary point of contact, and the GP assumes a coordinating and guiding role across providers and sectors. Participation in HzV is voluntary for patients, and the specific contractual arrangements vary between health insurers and regions.

A growing body of evidence demonstrates that GPCC (HzV) improves both quality of care and clinical outcomes for patients with HF compared with usual care [8–10]. The AOK Baden‐Württemberg HzV programme is a particularly established example of structured, team‐based primary care. Within this programme, GPs may be supported by Primary Care Practice Assistants (VERAH; Versorgungsassistentinnen und ‐assistenten in der Hausarztpraxis), specially trained nonphysician members of the general practice team.

VERAH (‘Versorgungsassistentin in der Hausarztpraxis’) are practice‐based healthcare professionals with additional training who can undertake delegated clinical and organisational activities within the scope of general practice. Depending on the contractual and organisational setting, their activities may include home visits, patient monitoring, telephone‐based follow‐up, care coordination, patient education and counselling and support for the management of patients with chronic conditions [11]. In the AOK Baden‐Württemberg HzV programme, VERAH‐related care for chronically ill patients is specifically incorporated into the contractual framework and separately documented for remuneration. Importantly, VERAH are not synonymous with GPCC itself: rather, they represent an additional nonphysician workforce component that can be integrated into GP‐centred primary care. For the purposes of this study, we refer to GPCC with extended multidisciplinary team involvement as ‘GPCC‐Plus’. GPCC‐Plus is an operational definition used in our study to distinguish practices providing GPCC with additional multidisciplinary team involvement, including VERAH‐supported care, from GPCC without this extended team component. Thus, GPCC‐Plus should not be understood as a separate nationwide statutory care model, but as the category used in this study to capture an enhanced form of GP‐centred primary care.

These functions are particularly relevant for multimorbid HF patients, who require frequent follow‐up and proactive management across care settings [12, 13]. The potential added value of VERAH therefore lies not in replacing GP‐led care, but in extending the capacity of the primary care team to provide structured follow‐up, coordination and delegated case‐management activities. This raises the question of whether GPCC with such additional team involvement provides incremental long‐term benefits beyond GPCC alone and usual care.

Multidisciplinary collaboration is also an established component of coordinated HF care. Collaboration between primary care, cardiology, nursing and other healthcare professionals can support continuity of care, patient self‐management, early recognition of clinical deterioration and timely intervention [9, 10, 14–16]. Previous evidence on nurse‐led and multidisciplinary interventions in HF suggests beneficial effects on hospital readmissions and health‐related quality of life, although evidence regarding mortality remains less consistent. These findings provide a broader clinical rationale for examining whether additional multidisciplinary workforce capacity within primary care is associated with improved long‐term outcomes [9, 10, 14–16].

While previous studies consistently show that GPCC outperforms uncoordinated usual care [17–19], evidence remains limited on the incremental long‐term benefit of integrating VERAH‐supported team care within an already coordinated GPCC model. In particular, it remains unclear whether the additional workforce capacity and delegated case‐management functions associated with VERAH translate into sustained reductions in hospitalisations and mortality among patients with HF.

This study therefore evaluates long‐term outcomes among patients with HF across three care categories: usual care, GPCC and GPCC‐Plus. We specifically examine whether GPCC‐Plus, characterised by additional VERAH‐supported team involvement, is associated with better long‐term outcomes than GPCC alone and usual care.

## 2. Methods

### 2.1. Study Design and Study Population

This retrospective cohort study evaluated the long‐term effects of structured, team‐based primary care among patients with HF over a 12‐year period (2011–2022) using routinely collected administrative claims data from Allgemeine Ortskrankenkasse (AOK) Baden‐Württemberg, one of the largest regional statutory health insurers in Germany, covering approximately 4.5 million insured individuals. All study groups were drawn from Baden‐Württemberg to ensure comparable healthcare structures across groups. Particular focus was placed on the contribution of delegated care management delivered by VERAH.

Patients with documented HF were identified using validated ICD‐10 codes from ambulatory and/or inpatient care records. In ambulatory care, only patients with confirmed diagnoses were included to ensure diagnostic validity, whereas inpatient HF diagnoses were based on hospital discharge diagnoses. An open‐cohort design with annual observation periods was applied, allowing repeated observations per individual for up to 12 years.

The study was conducted in accordance with the STROBE and STROSA guidelines. Ethical approval was obtained from the Ethics Committee of Frankfurt University Hospital (approval number 470/13).

### 2.2. Care Models and Group Comparison

This study compared three primary care models for patients with HF: usual care, GPCC and GPCC‐Plus. Tables 1 and 2 summarise the characteristics of the care models and the specific role of VERAH within GPCC‐Plus practices.

**TABLE 1:** Comparison of usual care vs. GP‐centred care (in Germany) [20].

Aspect	Usual care	GP‐centred care
Patient registration	No mandatory registration with a GP	Voluntary enrolment with a named GP

Care coordination	No structured gatekeeping: patients access specialists directly	Coordinated care with GP as gatekeeper (exceptions: gynaecology, ophthalmology, emergencies)

Remuneration	Fee‐for‐service with high documentation burden	Capitation and lump‐sum payments (e.g., for chronic care), reducing bureaucracy
		More time per patient due to simplified billing structure

Administrative burden	High due to extensive coding requirements	Reduced due to use of lump‐sum billing

Participation in DMP/QCs	Voluntary and not systematically implemented	Encouraged but not mandatory; integrated into care model

Quality circles^∗^/drug networks	Rarely institutionalised	Structured participation with feedback on prescribing and evidence‐based practice

Use of multidisciplinary team (VERAHs)	Not regularly implemented	Additional team‐based care, including VERAH®, may be implemented depending on the contractual and practice setting

Prevention and early detection	Depends on individual practice	Better integrated through continuous care and structured approaches

Collaboration with specialists	No formal feedback requirement	Structured coordination and referral processes within the GP‐centred care framework
		Contractually guaranteed shorter waiting times for enrolled patients
	Standard hours based on practice policies	Extended hours contractually required, including evening consultations
	Regular statutory co‐payments	Reduced co‐payments for selected medications and services

Target population	General population	Insured patients voluntarily enrolled in HzV; particularly relevant for patients with chronic and complex conditions

*Note:* DMP (disease management programme) is a structured, evidence‐based care programme for patients with specific chronic diseases. In Germany, separate DMPs exist for several chronic conditions, including diabetes, coronary heart disease, asthma, COPD and breast cancer; QCs–Quality Circles: Small groups of physicians that receive regular feedback on their prescribing behaviour, evidence‐based medical updates and support for implementing quality improvement measures.

**TABLE 2:** Comparison of usual care, GP‐centred care (GPCC) and GP‐centred care plus (GPCC‐plus), with a focus on multidisciplinary team‐based care.

Aspect	Usual care	GP‐centred care (GPCC)	GP‐centred care plus (GPCC‐plus)
Primary care coordination	No structured coordination	GP as coordinating provider	GP coordination plus additional structured team‐based coordination
Multidisciplinary team involvement	Not systematically defined	Not necessarily part of the model	Extended team involvement including Primary Care Practice Assistants (VERAH®)
VERAH® involvement	Not systematically implemented	May be available depending on practice and contractual arrangements	Defined VERAH®‐supported care identified through predefined AOK Baden‐Württemberg billing codes and lump‐sum payments
Case management	Mainly dependent on individual practice organisation	Primarily GP‐led	Delegated case‐management activities supported by VERAH®
Home visits	Practice‐dependent	Practice‐dependent	VERAH®‐supported home visits where applicable
Patient monitoring and follow‐up	Practice‐dependent	Primarily GP‐led	Additional structured monitoring and follow‐up by VERAH®
Care coordination across providers	Primarily organised by individual practice	Coordinated by GP	Shared between GP and VERAH® within the primary care team
Support for patients with chronic conditions	Practice‐dependent	Structured chronic care within GPCC	Additional team‐based support for patients with chronic and complex conditions
Role in the study	Reference care model	Coordinated primary care model	Extended multidisciplinary primary care model evaluated for additional long‐term benefit

GPCC is a structured primary care programme designed to improve coordination, continuity and quality of care for patients with chronic diseases. The programme includes structured chronic disease management, incentivised participation in nationwide disease management programmes (DMPs), active involvement of GPs in quality circles and medicines management networks, and implementation of evidence‐based primary care guidelines.

The main distinction between GPCC and GPCC‐Plus was the integration of Primary Care Practice Assistants (Versorgungsassistentin in der Hausarztpraxis, VERAH) into routine care delivery. Within GPCC‐Plus practices, VERAH supported delegated care processes including patient monitoring, medication counselling, structured follow‐up, home visits and elements of case management for chronically ill patients such as those with HF. Their involvement aimed to strengthen continuity of care, coordination across healthcare sectors, early recognition of clinical deterioration and patient self‐management support.

For operationalisation, VERAH integration was identified using claims‐based reimbursement data within the HzV billing system. HzV practices were classified as VERAH‐supported or non‐VERAH‐supported based on whether VERAH‐specific services were billed, using an established registry of participating HzV practices.

Patients enrolled voluntarily in the GPCC programme and selected one GP as their main coordinating provider. Specialist consultations generally occurred following GP referral, except for emergencies and routine visits to gynaecologists or ophthalmologists. Additional programme components included computerised prescribing support systems, structured cooperation with specialists and measures to improve access to primary care.

Usual care served as the reference group and reflected standard primary care without participation in the GPCC programme or structured integration of VERAH into routine care processes.

Table 1 illustrates the differences between usual care, GPCC and GPCC‐Plus, while Table 2 summarises the core responsibilities and activities of VERAH within GPCC‐Plus practices.

### 2.3. Adjustment Variables/Confounders

Potential confounders were identified a priori based on routinely available claims data and existing literature on HF and primary care utilisation (Am J Med. 2026; 139(6):692–701. doi:10.1016/j.amjmed.2026.02.017. Epub 2026 Mar 9). These included sociodemographic characteristics (age and sex), clinical comorbidities such as diabetes mellitus and coronary artery disease (CAD), as well as indicators of prior healthcare utilisation and overall disease burden. A comprehensive list of all variables included in the analysis is provided in Table 3 (Variable Legend).

**TABLE 3:** Characteristics of HF patients by group in 2011 (start of the study)^∗^.

Characteristics	Usual care	GPCC	GPCC‐plus	*p* value
Number of patients	38,551	23,192	20,770	
Socioeconomic parameters				
Mean age (years) (SD)	78.2 (10.3)	77.0 (10.0)	77.1 (10)	< 0.001
Sex (%) women	61.9	58.5	58.9	< 0.001
Need for long‐term care (%)	30.0	22.9	23.8	< 0.001
Rural practice (%)	45.6	46.1	41.5	< 0.001
Group practice	36.3	37.9	56.3	< 0.001
Health service utilisation (%)				
DMP CHD	11.7	21.3	26.2	< 0.001
DMP DM	20.5	31.3	35.1	< 0.001
Hospital admission due to CVD	15.8	15.2	14.9	0.006
Mean Charlson Comorbidity Index (SD)	3.6 (2.5)	3.6 (2.5)	3.8 (2.5)	< 0.001
Comorbid conditions (%)				
Renal failure	17.9	16.7	19.1	< 0.001
COPD	15.6	16.9	17.8	< 0.001
Dementia	10.3	7.8	8.7	< 0.001
Depression	19.3	17.5	17.7	< 0.001
Cardiovascular history (%)				
CHD	38.7	41.8	41.7	< 0.001
Myocardial infarction	1.3	1.2	1.1	0.283
Hypertension	75.9	76.0	78.4	< 0.001
Atrial fibrillation	26.9	26.6	26.1	0.126

*Note:* The following characteristics were used as potential confounders annually: Single‐physician practice: 1 GP; group practice: ≥ 2 GPs working in the same practice.

Abbreviations: CCI, Charlson Comorbidity Index; CHD, coronary heart disease; CHF, chronic heart failure; CKD, chronic kidney disease; COPD, chronic obstructive pulmonary disease; DM, diabetes mellitus; DMP, disease management program; SD, standard deviation.

These variables were included in all multivariable models to adjust for potential confounding and to minimise bias in the estimated effects across the different care models.

### 2.4. Outcomes

All data were recorded annually.

• All case hospitalisation was computed as at least one hospitalisation with at least one overnight stay in the year under observation, specified as a dichotomous outcome (1 = yes; 0 = no, ref.).
• Ambulatory care‐sensitive conditions (ACSCs) were calculated using ICD‐10 codes and the Purdy ACSC definition [21]. Positive ACSCs had to include at least one ACSC, indicated hospitalisation stay in the year under observation, specified as a dichotomous outcome (1 = yes; 0 = no, ref.).
• All case mortality was computed as a dichotomous outcome (1 = death; 0 = alive, ref.).

### 2.5. Statistical Analysis

Multivariable logistic regression models adjusted for confounders were applied annually over the 12‐year observation period. generalised estimating equations (GEEs) were used to account for repeated measures within patients. Intervention effects were expressed as odds ratios (ORs) compared with the control group, with time interaction terms. The interaction term indicates whether the effect of the intervention changes over time, with its corresponding OR reflecting the multiplicative difference in temporal trends between intervention and control groups. In addition, confidence intervals and *p* values were reported alongside all ORs. Marginal standard errors were calculated using GEE to obtain robust variance estimates for binomial outcomes. Predicted proportions derived from the models over time were computed for each of the 12 observation years and visualised graphically to illustrate longitudinal trends across study groups. To account for multiple testing, Bonferroni correction was applied. With 35 statistical tests performed, the overall significance level of *p* = 0.05 was adjusted to *p* = 0.0014 (0.05/35). All analyses were conducted using SAS Version 9.4 and IBM SPSS Statistics Version 25.

## 3. Results

At baseline, relevant differences were observed across the three study groups (Table 3). Patients in GPCC and GPCC‐Plus were slightly younger than those in usual care, with mean ages of 77.0 (SD 10.0) and 77.1 (SD 10.0) years, respectively, compared with 78.2 (SD 10.3) years in usual care. The proportion of women was lower in GPCC (58.5%) and GPCC‐Plus (58.9%) than in usual care (61.9%), and the need for long‐term care was also reduced in GPCC (22.9%) and GPCC‐Plus (23.8%) compared with usual care (30.0%). Healthcare organisation and utilisation patterns further differed across groups. GPCC‐Plus practices more frequently operated as group practices (56.3%) compared with GPCC (37.9%) and usual care (36.3%), while rural practice settings were slightly less common in GPCC‐Plus (41.5%) than in GPCC (46.1%) and usual care (45.6%). Participation in structured chronic disease management was higher in both GPCC models, with greater enrolment in disease management programmes for coronary heart disease (26.2% in GPCC‐Plus, 21.3% in GPCC and 11.7% in usual care) and diabetes mellitus (35.1%, 31.3% and 20.5% respectively). Comorbidity burden was slightly higher in GPCC‐Plus, as reflected by a mean Charlson Comorbidity Index of 3.8 (SD 2.5) compared with 3.6 (SD 2.5) in both GPCC and usual care. GPCC‐Plus patients also showed higher prevalences of renal failure (19.1%), COPD (17.8%) and hypertension (78.4%), while dementia (8.7%) and depression (17.7%) were slightly less frequent than in usual care (10.3% and 19.3% respectively). Cardiovascular history was broadly comparable across groups, including coronary heart disease (41.7% in GPCC‐Plus, 41.8% in GPCC and 38.7% in usual care).

Table 4 shows the effects of GPCC and GPCC‐Plus on outcomes in patients with HF over the observation period 2011–2022. At baseline, GPCC‐Plus was associated with significantly lower odds of all‐cause hospitalisation by 5.9% compared with both usual care (OR 0.941, 95% CI 0.923–0.959, *p* < 0.001) and 4,3% in GPCC (OR 0.957, 95% CI 0.937–0.977, *p* < 0.001). The longitudinal analysis demonstrated a consistent annual decline in hospitalisations across both GPCC models, with slightly stronger effects by 1,5% observed in GPCC‐Plus (OR per year 0.985, 95% CI 0.982–0.988).

**TABLE 4:** Results of multivariable logistic regression models for all outcomes among the GPCC and GPCC‐plus patients in comparison with usual care.

Outcome for observation period 2011–2022	Interpretation	Odds ratio	95% CI lower	95% CI upper	*p* value
All‐cause‐hospitalisation	GPCC‐Plus vs. usual care at baseline	0.941	0.923	0.959	< 0.001
	GPCC vs. usual care at baseline	0.983	0.963	1.004	0.112
	GPCC‐Plus vs. GPCC at baseline	0.957	0.937	0.977	< 0.001
	Usual care annually (time trend)	0.96	0.958	0.962	< 0.001
	GPCC‐Plus vs. usual care (time trend)	0.985	0.982	0.988	< 0.001
	GPCC vs. usual care (time trend)	0.988	0.984	0.991	< 0.001

Ambulatory care‐sensitive conditions (ACSC) hospitalisation	GPCC‐Plus vs. usual care at baseline	0.973	0.938	1.009	0.140
	GPCC vs. usual care at baseline	0.947	0.91	0.987	0.009
	GPCC‐Plus vs. GPCC at baseline	1.027	0.985	1.07	0.213
	Usual care annually (time trend)	0.96	0.956	0.964	< 0.001
	GPCC‐Plus vs. usual care (time trend)	0.977	0.972	0.983	< 0.001
	GPCC vs. usual care (time trend)	0.989	0.982	0.995	0.001

All‐cause mortality	GPCC‐Plus vs. usual care at baseline	0.929	0.899	0.96	< 0.001
	GPCC vs. usual care at baseline	1.119	1.08	1.159	< 0.001
	GPCC‐Plus vs. GPCC at baseline	0.83	0.8	0.862	< 0.001
	Usual care annually (time trend)	0.991	0.987	0.994	< 0.001
	GPCC‐Plus vs. usual care (time trend)	0.985	0.98	0.99	< 0.001
	GPCC vs. usual care (time trend)	0.984	0.979	0.99	< 0.001

For ACSC hospitalisations, no significant differences were observed at baseline for GPCC‐Plus; however, a gradual annual reduction was evident in both GPCC groups over time. All‐cause mortality was significantly lower by 7,1% in the GPCC‐Plus group at baseline (OR 0.929, 95% CI 0.899–0.960, *p* < 0.001) and showed a sustained downward trend over the study period. In contrast, GPCC showed no clear baseline advantage over usual care but demonstrated similar temporal improvements in mortality risk.

Overall, GPCC‐Plus—characterised by the integration of VERAH‐supported team‐based care—was associated with the most consistent long‐term reductions in hospitalisations and mortality among patients with HF.

Figures 1 and 2 display longitudinal trends in all‐cause and potentially avoidable hospitalisations between 2011 and 2022. Marginal annual percentages indicate persistently lower hospitalisation rates in the GPCC‐Plus group compared with GPCC and usual care.

**FIGURE 1:**
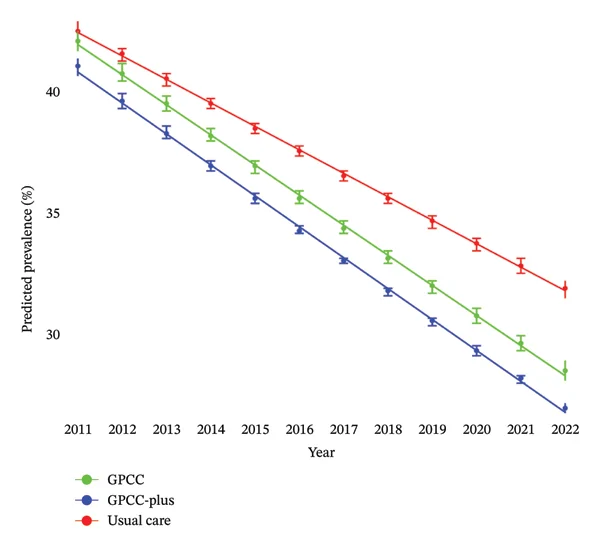
Marginal effects of estimated rates for all‐cause hospitalisation in heart failure patients.

**FIGURE 2:**
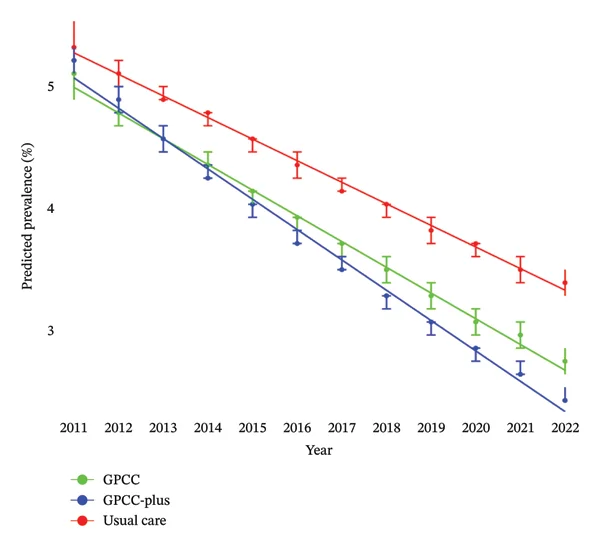
Marginal effects of estimated rates for ambulatory care‐sensitive conditions hospitalisation in heart failure patients.

Figure 3 illustrates longitudinal trends in all‐cause mortality over the study period. Despite heterogeneity across years, GPCC‐Plus consistently showed lower mortality rates, particularly during the COVID‐19 pandemic period from 2020 onwards, whereas differences between GPCC and usual care were less pronounced.

**FIGURE 3:**
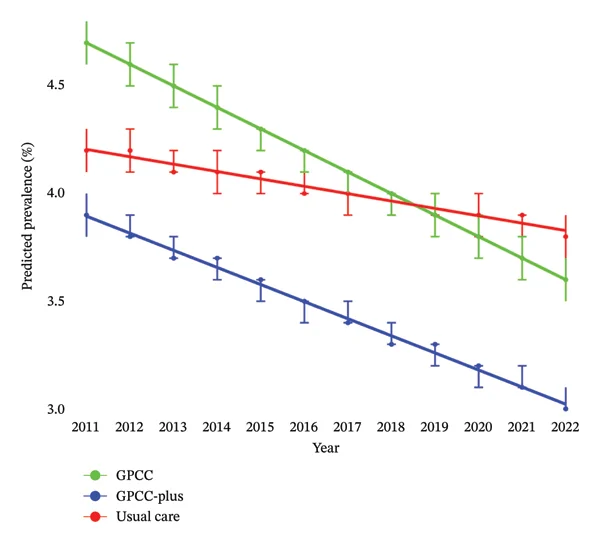
Marginal effects of estimated rates for all‐cause mortality in heart failure patients.

## 4. Discussion

Our findings indicate that the effectiveness of coordinated primary care extends beyond physician continuity alone and critically depends on the integration of trained nonphysician staff within structured team‐based care models. In particular, the observed benefits of GPCC are likely driven not only by the GP as coordinating provider but also by systematic task delegation to Primary Care Practice Assistants (VERAH). For complex and resource‐intensive conditions such as HF, delegated case management may play an important role in reducing preventable hospitalisations and improving survival outcomes.

Within this context, VERAH‐supported care represents a distinctive implementation of multidisciplinary management in primary care, as Primary Care Practice Assistants (VERAH) operationalise structured care processes directly within the GP practice rather than through external coordination systems. A large systematic review of randomised trials shows that multidisciplinary HF programmes reduce hospitalisations and, when specialist follow‐up is included, also reduce mortality—supporting the effectiveness of structured team‐based care [22]. However, a more recent meta‐analysis found that multidisciplinary programmes initiated outside primary care, particularly those implemented solely in community settings without GP integration, did not reduce mortality or readmissions, unlike hospital‐initiated programmes. This highlights the need to strengthen and evaluate multidisciplinary models that are fully integrated into GP‐led primary care structures [23].

International evidence on integrated primary care and multimorbidity further supports the importance of coordinated, patient‐centred care. The 3D study, published in ∗The Lancet∗, reported on a pragmatic cluster‐randomised trial evaluating a patient‐centred model of care for people with multimorbidity in primary care and highlighted the complexity of implementing integrated approaches across multiple chronic conditions [19]. Although the intervention did not significantly improve the primary quality‐of‐life outcome, the study illustrates the importance of coordinated primary care for patients with multimorbidity and provides an international context for our findings.

Our findings extend this evidence by focusing specifically on patients with HF and by comparing GPCC with and without extended VERAH‐supported multidisciplinary team involvement over a 12‐year period.

An umbrella review showed that nurse‐led and multidisciplinary chronic disease self‐management interventions in HF are consistently associated with reduced hospital readmissions and improved health‐related quality of life, while evidence on mortality reduction remains mixed. Key gaps include limited cost‐effectiveness analyses, insufficient consideration of culturally tailored models and heterogeneous outcome reporting, highlighting the need for more standardised and health‐economic evaluations [24].

Our findings extend this evidence by suggesting that structured delegation through Primary Care Practice Assistants (VERAH) may represent a key mechanism through which multidisciplinary care is translated into routine ambulatory practice. VERAH support proactive follow‐up, home visits, medication counselling, structured monitoring and early detection of clinical deterioration, thereby strengthening continuity and coordination of care for patients with HF.

Our results are consistent with broader evidence on sustainable HF management in primary care [22, 23, 25, 26]. Older and frail patients may not benefit from extensive specialist investigations or aggressive interventions, underscoring the importance of patient‐centred approaches that account for multimorbidity and functional status [1]. In this regard, Primary Care Practice Assistants (VERAH) may be particularly relevant, as they enable continuous patient contact, structured monitoring and early intervention outside physician consultations.

The baseline characteristics in our study provide additional context for interpreting these findings. GPCC‐Plus patients were slightly younger and had a lower need for long‐term care than patients receiving usual care, but they also had a slightly higher overall comorbidity burden and higher prevalences of renal failure, COPD and hypertension. Thus, GPCC‐Plus patients were not uniformly a lower‐risk population. The higher proportion of group practices and greater participation in structured disease management programmes in GPCC‐Plus may reflect organisational characteristics that facilitate multidisciplinary and structured care.

The biggest differences between the care models were observed for all‐cause hospitalisation. Because GPCC and GPCC‐Plus both involve GPCC, the additional difference between these groups is particularly relevant and suggests that extended multidisciplinary team involvement may provide benefits beyond GP coordination alone.

For ACSC hospitalisations, no significant baseline difference was observed for GPCC‐Plus. However, a gradual annual reduction was evident in both GPCC groups. This pattern indicates that the potential benefits of coordinated primary care may become more apparent over time rather than being reflected exclusively in baseline differences.

The mortality findings provide further support for the potential long‐term relevance of extended multidisciplinary primary care. GPCC‐Plus was associated with 7.1% lower odds of all‐cause mortality at baseline and showed a sustained downward trend over the observation period. GPCC did not show a clear baseline advantage over usual care but demonstrated similar temporal improvements in mortality risk. The consistently lower mortality observed in GPCC‐Plus suggests a potentially important association between extended team‐based primary care and long‐term outcomes. However, given the observational design, these findings should not be interpreted as evidence of a causal effect of VERAH involvement. Overall, GPCC‐Plus—characterised by the integration of VERAH‐supported team‐based care—was associated with the most consistent long‐term reductions in hospitalisations and mortality among patients with HF. This finding is particularly relevant because the comparison with GPCC provides an assessment of the potential additional contribution of extended multidisciplinary team involvement within an already coordinated primary care model.

The organisational context may also be relevant. GPCC‐Plus practices were more frequently organised as group practices than GPCC and usual‐care practices. This may indicate that practice‐level capacity for task sharing and multidisciplinary collaboration facilitates the implementation of VERAH‐supported care. However, claims data cannot determine whether the observed differences were attributable to VERAH activities themselves or to broader organisational characteristics of participating practices.

Taken together, our findings support further evaluation of VERAH‐supported multidisciplinary primary care as an integrated workforce model for patients with HF. Future studies should investigate which specific VERAH activities contribute most to improved outcomes, as well as the implementation requirements, resource use and cost‐effectiveness of this approach.

### 4.1. Limitations

A major limitation of our study is the insufficient specificity of HF coding, which does not reliably capture clinical subtypes, underlying cardiac pathology, or corresponding pharmacotherapy. Because accurate assessment of treatment quality is not possible without correct diagnostic codes, medication‐related analyses were deliberately excluded. Our analyses therefore focused on clinical outcomes rather than pharmacological treatment patterns or treatment quality.

This study relies on claims data, which do not capture clinical parameters or directly observe the specific activities performed by VERAH. Enrolment in GPCC and practice‐level indicators were used as proxies for exposure to delegated case management. Residual confounding and potential misclassification cannot be excluded. Additional sources of selection bias may exist, such as differences in patient or physician engagement with GPCC. To mitigate confounding, we included and adjusted for all available socioeconomic factors and clinical risk factors obtainable from the data. Beyond these adjustments, the claims data do not allow further control for unmeasured confounders or behavioural factors.

Importantly, the claims data do not allow us to determine which specific VERAH activities were performed for individual patients or how frequently these activities were delivered. Therefore, the observed differences between GPCC and GPCC‐Plus should be interpreted as associations with the extended care model rather than as direct effects of individual VERAH interventions.

These limitations notwithstanding, the 12‐year observation period and the consistent longitudinal patterns observed in GPCC‐Plus strengthen the relevance of the findings for long‐term primary care organisation.

## 5. Conclusion

The findings of this study highlight the long‐term benefits of GPCC delivered in collaboration with an extended multidisciplinary team. In particular, structured and delegated case management provided by Primary Care Practice Assistants (VERAH) enables continuous monitoring, early detection of clinical deterioration and coordinated care across healthcare sectors. GPCC‐Plus was associated with the most consistent reductions in hospitalisation and mortality over the 12‐year observation period, including an additional reduction in hospitalisation compared with GPCC. This integrated primary care model, in which VERAH plays a central operational role in routine chronic disease management, is associated with reduced hospitalisations and improved survival among patients with HF.

Given these findings, future research should focus on identifying the specific components of VERAH‐supported care that are most effective and on evaluating the feasibility, resource requirements and cost‐effectiveness of expanding this multidisciplinary primary care model.

## Funding

The evaluation of GP‐centred healthcare in the German regional state of Baden‐Württemberg was funded by AOK Baden‐Württemberg, Association of General Practitioners Baden‐Württemberg (Hausärzteverband und HÄVG) and MEDI Baden‐Württemberg. Open Access funding was enabled and organised by Projekt DEAL.

## Ethics Statement

Ethical approval was granted by the University Hospital Frankfurt Medicine Ethics Committee (reference number: 430/13).

## Conflicts of Interest

The authors declare no conflicts of interest.

## Supporting Information

Additional supporting information can be found online in the Supporting Information section.

## Supporting information


**Supporting Information** STROBE checklist.

## Data Availability

The data supporting the findings of this study are owned by a statutory health insurance provider in Germany and cannot be made publicly available due to data protection and legal restrictions. However, the authors are happy to provide the analysis scripts, code, and other relevant methodological materials upon reasonable request to the corresponding author.
